# High-level ePVS was accompanied by an increase in kidney transplant failure risk: analysis based on the MIMIC-IV database

**DOI:** 10.3389/fimmu.2025.1574525

**Published:** 2025-08-29

**Authors:** Zhirong Zhou, Lin Zhang, Delin Zhang, Yan Yang, Shuiping Ou

**Affiliations:** ^1^ Department of Pharmacy, Affiliated Hospital of Zunyi Medical University, Zunyi, Guizhou, China; ^2^ First Teaching Hospital of Tianjin University of Traditional Chinese Medicine, Tianjin, China

**Keywords:** ePVS, estimated plasma volume status, PV, plasma volume, kidney transplant failure, MIMIC-IV database

## Abstract

**Background:**

The prognosis of kidney transplantation is currently assessed primarily through clinical monitoring, which involves considerable time and financial costs. Estimated plasma volume status (ePVS) has emerged as a straightforward and efficient method for evaluating patient condition. However, the potential prognostic significance of ePVS in kidney transplant recipients has yet to be thoroughly investigated.

**Methods:**

The clinical data for the patient were obtained from the MIMIC-IV database. ePVS was calculated based on hematocrit and hemoglobin values upon admission. Baseline characteristics were compared according to ePVS quartiles, and the relationship between ePVS levels and kidney transplant failure (KTF) in patients was assessed using a Logistic regression model.

**Results:**

4,421 eligible subjects (2,584 males and 1,837 females) with an average age of 52.53 ± 13.00 years old were included in our study. 3,661 (82.80%) had no kidney transplant failure (No-KTF) and 760 (17.20%) had kidney transplant failure (KTF). The ePVS values exhibited a skewed distribution, with the admission patients concentrated in the range of 4–8 mL/g and the discharge patients concentrated in the range of 6–10 mL/g. The ePVS level in the KTF group (7.20 [5.78, 8.85]) was significantly higher than that in the non-KTF group (6.12 [4.95, 7.60]) (*p*< 0.001) at admission. The ePVS level in the KTF group (8.18 [6.71, 9.47]) was significantly higher than that in the non-KTF group (7.01 [5.56, 8.55]) (*p*< 0.001) at discharge. The sensitivity values were 0.851 and 0.805, the specificity values were 0.744 and 0.81, and the AUC values were 0.861 and 0.847, respectively, at admission and discharge. In our subgroup analysis, including interactive validation, we found that regardless of admission or discharge, the risk of KTF was greater when ePVS increased in Non-heart failure (HF) (*P*-interaction<0.001).

**Conclusion:**

In this study, we found that higher ePVS values were accompanied by an increase in KTF risk, and this association proved robust and independent of age, gender, and comorbidities. Additionally, in our subgroup analysis, including interactive validation, we found that regardless of admission or discharge, the risk of KTF was greater when ePVS increased in non-heart failure. Therefore, ePVS may be an important reference parameter for kidney transplant patients and help improve risk stratification.

## Introduction

Kidney transplantation was widely regarded as the preferred treatment for patients with end-stage renal disease, as successful transplantation was linked to longer survival and a better quality of life when compared to dialysis. Alloreactive immune responses against the donor’s kidney can lead to acute rejection of the transplant. Currently, the prognosis of kidney transplantation was primarily assessed through clinical monitoring, including serum creatinine levels, proteinuria, and histopathologic evaluation of kidney transplant biopsies. Identifying and validating biomarkers that correlate with or predict acute rejection was a priority for the transplantation community, as these could enhance therapeutic decision-making. However, these diagnostic tests require substantial time and financial resources. Therefore, there is an urgent need for a rapid, simple, and minimally invasive method to assist in evaluating the prognosis of kidney transplant patients.

The traditional Strauss et al. formula, developed in 1951, uses an equation based on hematocrit and hemoglobin to estimate plasma volume status (ePVS) (1). In 2015, Duarte et al. introduced a single time-point “instantaneous” measurement of plasma volume, derived from the Strauss formula, for estimating PV (2). They found that, in cases of myocardial infarction complicated by heart failure (HF). ePVS provides a simple method for estimating plasma volume. As a surrogate marker for total vascular volume, it has been validated for its reliability, with multiple studies showing its independent association with outcomes across various heart failure phenotypes (3, 4). Moreover, beyond cardiovascular disease, ePVS has proven to be an effective tool for assessing disease prognosis in conditions such as infectious shock, lower limb arterial disease, thrombosis, and other illnesses (5, 6).

Recent studies have indicated an association between ePVS or PV and kidney injury. In a retrospective cohort study, ePVS was found to be a promising parameter for assessing the risk of acute kidney injury (AKI) in patients undergoing coronary revascularization (7). Additionally, a higher PVS was linked to an increased incidence of new-onset AKI and poorer outcomes in a cohort of hospitalized COVID-19 patients (8). Furthermore, a decrease in estimated plasma volume during hospitalization serves as a predictive indicator of renal function deterioration in acute heart failure (9). However, to the best of our knowledge, the relationship between ePVS and the risk of kidney transplant failure (KTF) in renal transplant recipients remains unclear.

This study aimed to investigate the association between ePVS and kidney transplant failure (KTF) in renal transplant patients, and whether this association persists across different age groups, genders, and complications. The findings may provide valuable insights for risk stratification and management in kidney transplant recipients.

## Methods

### Data source

The Medical Information Mart for Intensive Care IV (MIMIC-IV version 3.1) database was utilized to gather the data for this investigation (10). The MIMIC-IV database collected clinical data on patients who visited Beth Israel Deaconess Medical Center (BIDMC) between 2008 and 2019.

### Population selection

We included patients diagnosed with Complications of kidney transplant (codes T861), Unspecified complication of kidney transplant (codes T8610), Kidney transplant rejection (codes T8611), Kidney Transplant Failures (codes T8612), Kidney transplant infection (codes T8613), Other complication of kidney transplant (codes T8619), Encounter for aftercare following kidney transplant (codes Z4822) and Kidney transplant status (codes Z940) at hospital admission by the International Classification of Diseases (ICD)-9 diagnosis in the MIMIC-IV database. All patients aged > 18 years old.

The exclusion criteria were: (1) Age ≤18 years old, (2) Non-kidney transplant diagnosis; (3) Missing red blood cell volume and hemoglobin; (4) During pregnancy and the postpartum period; (5) Duration of hospital stay< 24 h; (6) Incomplete or unobtainable documented or other vital medical data records; (7) missing survival outcome data.

### Variable extraction

Data acquisition: The baseline characteristics of patients include age, gender, length of stay (LOS), history of Heart Failure, Diabetes Mellitus, CAD, Hypertension, Hyperlipidemia, Atrial Fibrillation, Anemia, Depressive, Hyperuricemia, and Obstructive Sleep Apnea. Therapeutic drugs include Insulin, Tacrolimus, Mycophenolate, Furosemide, Heparin, Prednisone, Warfarin, Vancomycin, Hydromorphone, Oxycodone, and Aspirin. Laboratory parameters include Basophils (BAS), Eosinophils, Lymphocytes, Monocytes(MO), Neutrophils, Anion Gap, Hematocrit, Hemoglobin, International Normalized Ratio (INR), Mean Corpuscular Hemoglobin (MCH), Mean Corpuscular Hemoglobin Concentration (MCHC), Mean Corpuscular Volume (MCV), Platelet (PLT), prothrombin time (PT), Red Cell Distribution Width (RDW), Red Blood Cell (RBC), White Blood Cell count (WBC), Bicarbonate, Calcium, Chloride(CL), Creatinine(Cr), Glucose, Magnesium, Phosphate, Potassium, Sodium, blood urea nitrogen (BUN).

### Missing data handling

Variables with more than 19% missing are deleted, while the remaining missing data are complemented by multiple imputation in the mice package, with the interpolation method pmm.

### Evaluation of ePVS

The Duarte formula incorporating hematocrit and hemoglobin was utilized as follows (1):


ePVS (dL/g)=(100-hematocrit (%))/hemoglobin (g/dL)

### Statistical analysis

Continuous variables with a normal distribution are presented as mean ± standard deviation (SD), and between-group differences were compared using the Student’s t-test or Satterthwaite t-test. For continuous variables with a skewed distribution, data are presented as medians and interquartile ranges (M [Q1, Q3]) and compared using the Mann-Whitney U-test. Categorical variables are expressed as frequencies and percentages [n (%)], and the Chi-square test or Fisher’s exact test was used to compare differences between the groups. Variables based on epidemiological, therapeutic drugs, and laboratory test indicators may serve as potential confounders (11). Therefore, four logistic regression models were employed to adjust for these potential confounders. In Model I, covariates were primarily adjusted for vital signs data (age, gender, LOS). In Model II, covariates were adjusted for comorbidities (CAD, HF, DM, HTN, AF, Anemia, OSA) in addition to those in Model I. In Model III, covariates were adjusted for therapeutic drugs tacrolimus, mycophenolate, furosemide, heparin, prednisone, warfarin, vancomycin, hydromorphone, and oxycodone based on Model II. In Model IV, covariates were further adjusted for laboratory data (INR, WBC, anion gap, calcium, chloride, platelet) in addition to those in Model III.

Univariate and multivariate Logistic regression models were used to assess the association between ePVS and kidney transplant failure (KTF) in patients who had undergone kidney transplantation. The associations were further explored across different subgroups based on age, gender, and medical history (CAD, HF, DM, HTN, AF, Anemia, and OSA), including admission and discharge. The results were expressed as odds ratios (OR) with 95% confidence intervals (CIs). All tests were two-tailed, with P ≤0.05 considered statistically significant. Statistical analyses were performed using SAS 9.4 (SAS Institute Inc., Cary, NC, USA) and R version 4.2.2 (Institute for Statistics and Mathematics, Vienna, Austria). Statistical significance was determined when the P-value was< 0.05.

## Results

### The characteristics of ePVS

**Figure 1:**
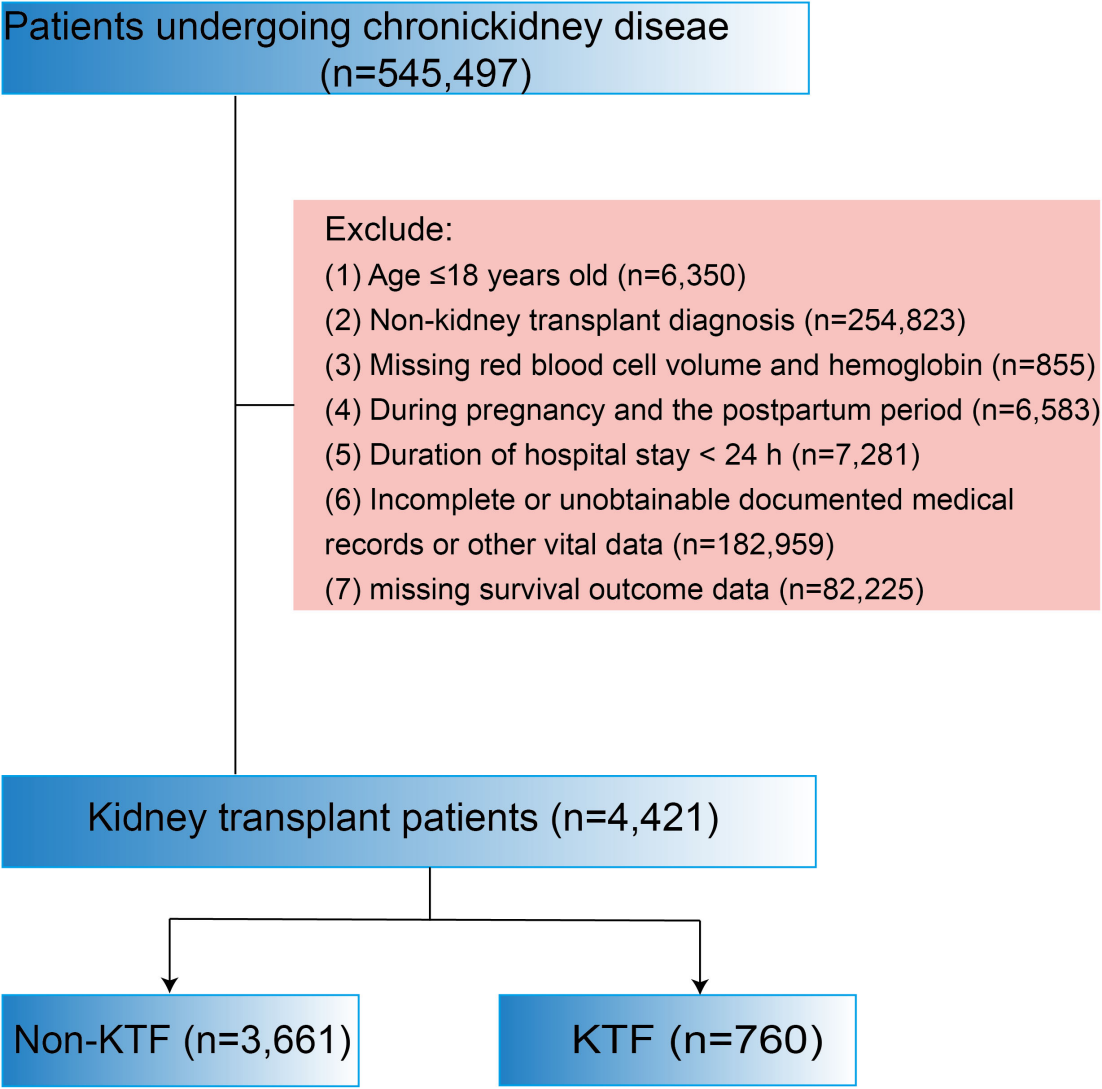
Flow chart of the study.

**Figure 2:**
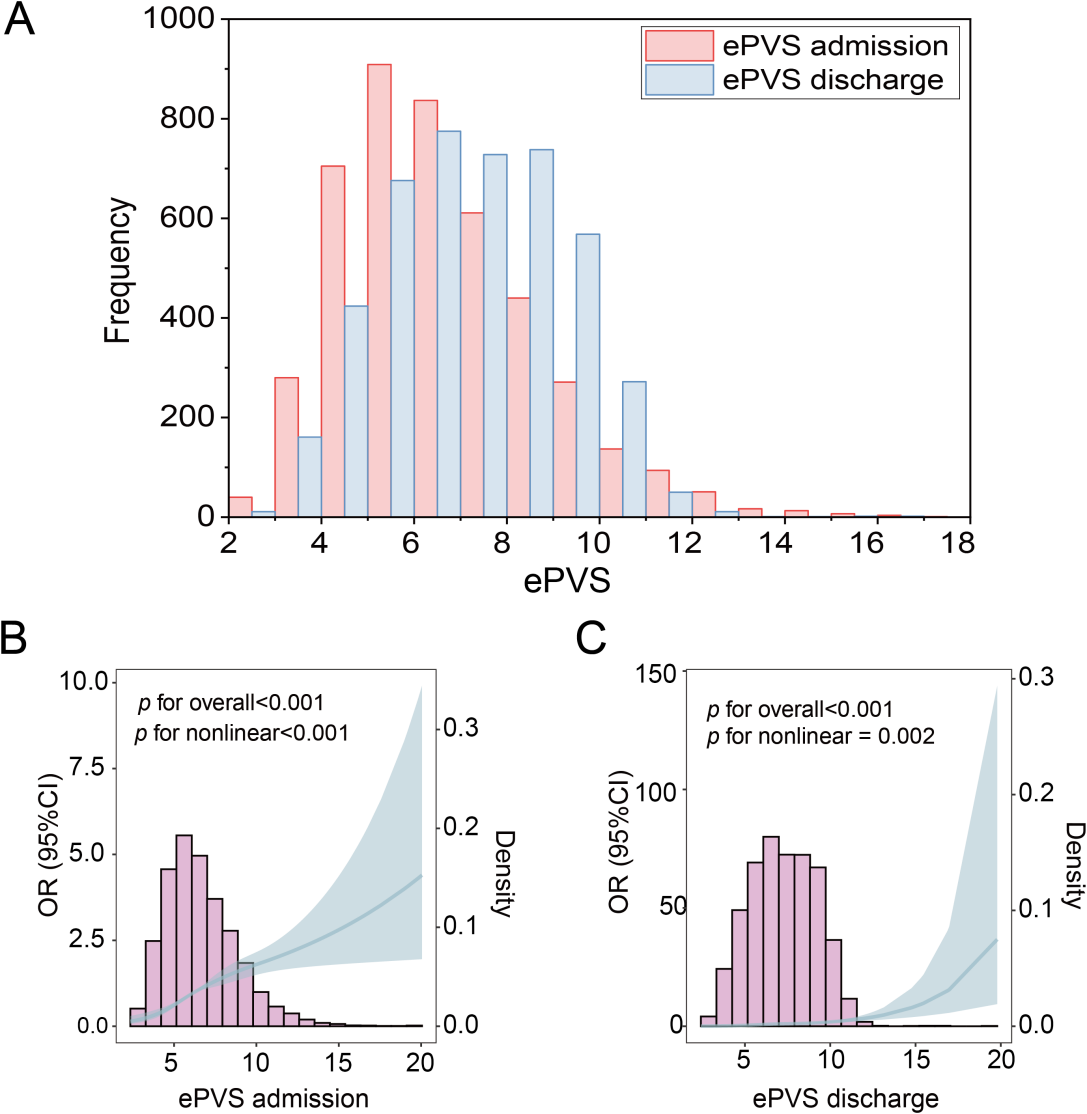
Distribution of ePVS in the entire study. **(A)** ePVS frequency distribution histogram, admission and discharge. **(B)** Restricted cubic spline (RCS) of admission patients. **(C)** Restricted cubic spline (RCS) of discharge patients. ePVS, estimated plasma volume status.

The flowchart of study participants was presented in **Figure 1**. We excluded 6,350 patients aged ≤18 years, 7,281 patients with a hospital stay of<24 hours, 254,823 patients with non-kidney transplant diagnosis, 855 patients missing red blood cell volume and hemoglobin data, 6,583 patients diagnosed with pregnancy or in the postpartum period, 182,959 patients with incomplete or unobtainable medical records or other vital data, and 82,225 patients with missing survival outcome data. Ultimately, 4,421 kidney transplant patients were included, of which 3,661 (82.80%) had no kidney transplant failure (No-KTF) and 760 (17.20%) had kidney transplant failure (KTF). The distribution of ePVS is shown in **Figure 2A**. The ePVS values exhibited a skewed distribution, with the admission patients concentrated in the range of 4–8 mL/g and the discharge patients concentrated in the range of 6–10 mL/g. The relationship between the admission ePVS level and kidney transplant failure (KTF) was nonlinear; higher ePVS levels were associated with an increased risk of kidney transplant failure (**Figure 2B**). Surprisingly, although discharged patients still have a high level of ePVS, their chance of acquiring KTF is significantly reduced (**Figure 2C**). This suggests that patients who have not yet undergone kidney transplantation may be more appropriate for evaluating the correlation between ePVS and KTF.

### The characteristics of study patients

A total of 4,421 kidney transplant patients were included, with 2,584 males and 1,837 females (**Table 1**). The mean age of all patients was 52.53 ± 13.00 years. The patients for ePVS quartile distribution were as follows: 4.37 dL/g (3.90-4.78) for the first quartile (Q1), 5.65 dL/g (5.39-5.99) for the second quartile (Q2), 6.98 dL/g (6.60-7.38) for the third quartile (Q3), and 9.16 dL/g (8.43-10.27) for the fourth quartile (Q4). There were a total of 1108 patients in the first quadrant(Q1), followed by 92 in the KTF group and 1016 in the non-KTF group; There were a total of 1108 patients in the first quadrant(Q2), 7followed by 168 in the KTF group and 940 in the non-KTF group; There were a total of 1100 patients in the first quadrant(Q3), followed by 205 in the KTF group and 895 in the non-KTF group; There were a total of 1105 patients in the first quadrant(Q4), followed by 295 in the KTF group and 810 in the non-KTF group. Obviously, high-level ePVS were accompanied by an increase in KTF risk.

**Table 1:** Characteristics of patients who have performed kidney transplantation.

Characteristic	Overall N = 4,421	True N = 760	False N = 3,661	*p*-value
Sex				0.054
F	1,837 (42%)	292 (38%)	1,545 (42%)	
M	2,584 (58%)	468 (62%)	2,116 (58%)	
Age	53 (45, 62)	52 (45, 61)	53 (44, 62)	0.2
LOS	4 (2, 8)	5 (3, 10)	4 (2, 7)	<0.001
Kidney Transplant Rejection	295 (6.7%)	27 (3.6%)	268 (7.3%)	<0.001
Kidney Transplant Infection	164 (3.7%)	17 (2.2%)	147 (4.0%)	0.018
Heart Failure	1,304 (29%)	319 (42%)	985 (27%)	<0.001
Diabetes Mellitus	2,433 (55%)	386 (51%)	2,047 (56%)	0.010
CAD	1,664 (38%)	334 (44%)	1,330 (36%)	<0.001
Hypertension	1,671 (38%)	184 (24%)	1,487 (41%)	<0.001
Hyperlipidemia	2,139 (48%)	347 (46%)	1,792 (49%)	0.10
Atrial Fibrillation	951 (22%)	210 (28%)	741 (20%)	<0.001
Anemia	2,103 (48%)	589 (78%)	1,514 (41%)	<0.001
Depressive	746 (17%)	136 (18%)	610 (17%)	0.4
Hyperuricemia	748 (17%)	143 (19%)	605 (17%)	0.13
Obstructive Sleep Apnea	577 (13%)	124 (16%)	453 (12%)	0.003
Insulin	2,373 (54%)	416 (55%)	1,957 (53%)	0.5
Tacrolimus/Mycophenolate	3,529 (80%)	375 (49%)	3,154 (86%)	<0.001
Furosemide	1,093 (25%)	156 (21%)	937 (26%)	0.003
Heparin	3,701 (84%)	693 (91%)	3,008 (82%)	<0.001
Prednisone	2,691 (61%)	438 (58%)	2,253 (62%)	0.045
Warfarin	694 (16%)	177 (23%)	517 (14%)	<0.001
Vancomycin	1,371 (31%)	329 (43%)	1,042 (28%)	<0.001
Hydromorphone	1,029 (23%)	249 (33%)	780 (21%)	<0.001
Oxycodone	1,457 (33%)	302 (40%)	1,155 (32%)	<0.001
Aspirin	2,281 (52%)	412 (54%)	1,869 (51%)	0.11
Anion Gap	15.0 (13.0, 18.0)	18.0 (15.0, 21.0)	15.0 (13.0, 18.0)	<0.001
Basophils (%)	0.30 (0.20, 0.50)	0.40 (0.20, 0.60)	0.30 (0.20, 0.50)	<0.001
Bicarbonate (mEq/L)	22.0 (20.0, 25.0)	23.0 (20.0, 26.0)	22.0 (20.0, 25.0)	<0.001
Calcium (mg/dL)	9.10 (8.60, 9.60)	8.80 (8.30, 9.40)	9.20 (8.70, 9.60)	<0.001
Chloride (mEq/L)	101 (97, 104)	96 (93, 100)	101 (98, 105)	<0.001
Creatinine (mg/dL)	1.90 (1.30, 3.60)	5.40 (3.30, 7.80)	1.60 (1.20, 2.60)	<0.001
Eosinophils (%)	0.80 (0.20, 2.10)	1.40 (0.40, 3.25)	0.80 (0.20, 2.00)	<0.001
Glucose (mg/dL)	122 (99, 179)	114 (92, 167)	123 (100, 182)	<0.001
Hematocrit (%)	34 (29, 38)	31 (27, 36)	34 (30, 39)	<0.001
Hemoglobin (g/Dl)	10.60 (9.00, 12.20)	9.60 (8.30, 11.10)	10.70 (9.20, 12.40)	<0.001
INR	1.20 (1.10, 1.40)	1.20 (1.10, 1.50)	1.20 (1.10, 1.40)	<0.001
Lymphocytes (%)	11 (6, 18)	12 (7, 19)	11 (6, 18)	0.001
MCHC (%)	31.60 (30.70, 32.60)	31.30 (30.40, 32.20)	31.70 (30.80, 32.60)	<0.001
MCH (%)	29.30 (27.60, 31.00)	29.90 (28.00, 31.60)	29.20 (27.50, 30.90)	<0.001
MCV (%)	93 (88, 97)	95 (90, 100)	92 (87, 97)	<0.001
Magnesium (mg/dL)	1.80 (1.60, 2.10)	2.10 (1.80, 2.30)	1.80 (1.60, 2.00)	<0.001
Neutrophils (%)	78 (68, 85)	75 (66, 84)	78 (68, 85)	<0.001
PT (sec)	13 (11, 15)	13 (12, 16)	13 (11, 15)	<0.001
Phosphate (mg/dL)	3.50 (2.80, 4.30)	4.60 (3.50, 5.80)	3.30 (2.70, 4.00)	<0.001
Platelet	202 (156, 262)	196 (157, 256)	204 (156, 264)	0.077
Potassium (mEq/L)	4.60 (4.20, 5.10)	4.70 (4.20, 5.50)	4.60 (4.20, 5.10)	<0.001
RDWCV (%)	14.90 (13.70, 16.50)	15.90 (14.50, 17.70)	14.60 (13.50, 16.20)	<0.001
Red Blood Cells (hpf)	3.63 (3.07, 4.25)	3.24 (2.78, 3.79)	3.72 (3.16, 4.31)	<0.001
Sodium (mEq/L)	138.0 (135.0, 140.0)	137.0 (134.0, 139.0)	138.0 (135.0, 140.0)	<0.001
BUN (mmol/L)	31 (20, 49)	47 (31, 65)	28 (19, 45)	<0.001
White Blood Cells (hpf)	7.7 (5.6, 10.4)	8.2 (6.0, 11.1)	7.6 (5.5, 10.3)	<0.001
ePVS_admission	6.27 (5.09, 7.88)	7.20 (5.78, 8.85)	6.12 (4.95, 7.60)	<0.001
ePVS_discharge	7.21 (5.75, 8.75)	8.18 (6.71, 9.47)	7.01 (5.56, 8.55)	<0.001
ePVS_delta	0.58 (0.00, 1.36)	0.68 (-0.15, 1.56)	0.57 (0.00, 1.33)	0.3
eGFR_admission	34 (16, 54)	10 (7, 18)	40 (23, 57)	<0.001
eGFR_discharge	40 (19, 61)	12 (8, 19)	46 (27, 65)	<0.001
eGFR_delta	3 (0, 10)	1 (-1, 5)	3 (0, 11)	<0.001
ePVSQ				<0.001
Q1 (3.90-4.78 dL/g)	1,108 (25%)	92 (12%)	1,016 (28%)	
Q2 ((5.39-5.99 dL/g)	1,108 (25%)	168 (22%)	940 (26%)	
Q3 (6.60-7.38 dL/g)	1,100 (25%)	205 (27%)	895 (24%)	
Q4 (8.43-10.27 dL/g)	1,105 (25%)	295 (39%)	810 (22%)	

^1^n(%); Median (Q1, Q3).

^2^Pearson’s Chi-squared test; Kruskal-Wallis rank sum test.

The ePVS level in the KTF group (7.20 [5.78, 8.85]) was significantly higher than that in the non-KTF group (6.12 [4.95, 7.60]) (*p*< 0.001) at admission. Similarly, the ePVS level in the KTF group (8.18 [6.71, 9.47]) was significantly higher than that in the non-KTF group (7.01 [5.56, 8.55]) (*p*< 0.001) at discharge. Furthermore, estimated glomerular filtration rate (eGFR), an important metric for evaluating renal function, was included in our research. The eGFR level in the KTF group (10 [7, 18]) was significantly lower than that in the non-KTF group (40 [23, 57]) (*p*< 0.001) at admission. Until discharge, this pattern was still maintained (KTF group (12 [8, 19]), non-KTF group (46 [27, 65]), *p*< 0.001).

Significant differences were observed between the two groups in terms of various parameters, including BAS, eosinophils, hematocrit, hemoglobin, lymphocytes, MCHC, INR, MCV, neutrophils, PT, RBC, WBC, bicarbonate, calcium, chloride, creatinine, glucose, magnesium, phosphate, potassium, sodium, and BUN (all *p*< 0.001). The comorbidities of kidney transplant patients in the KTF and non-KTF groups showed significant differences between the groups regarding heart failure, diabetes mellitus, CAD, hypertension, hyperlipidemia, atrial fibrillation, anemia, and obstructive sleep apnea (all *p* ≤ 0.01). In particular, patients with heart failure (42% *vs* 27%), CAD (44% *vs* 36%), atrial fibrillation (28% *vs* 20%), anemia (78% *vs* 41%) and obstructive sleep apnea(16% *vs* 12%) were more prone to KTF. In addition, male patients were often more prone to KTF events than female patients, and the LOS in the KTF group was significantly longer than that in the non-KTF group.

### Association between ePVS and the odds of KTF in patients who underwent kidney transplantation

**Table 2** depicts the relationship between ePVS and the risk of KTF in patients who underwent the underwent kidney transplantation at admission and discharge. When each unit of ePVS was increased, the risk of KTF in patients received kidney transplantation increased by 0.11 times (OR=1.11, *P*< 0.001) at admission, after adjustments for age, gender, LOS, CAD, HF, DM, HTN, AF, Anemia, OSA, tacrolimus, mycophenolate, furosemide, heparin, prednisone, warfarin, vancomycin, hydromorphone, oxycodone, INR, WBC, anion gap, calcium, chloride, and platelet. In discharged patients, when adjusted for the same confounders, the each unit of ePVS was increased, the risk of KTF in patients received kidney transplantation increased by 0.14 times (OR = 1.14, *P*< 0.001). Overall, if a kidney transplant patient has a high ePVS, then it suggests a greater chance of developing KTF.

**Table 2:** Association of ePVS and KTF of kidney transplantation.

Characteristic	Model	OR (95%CI)	*p*-value
ePVS_admission	Unadjusted Model	1.22 (1.18, 1.26)	<0.001
	Model I	1.22 (1.18, 1.26)	<0.001
	Model II	1.09 (1.05, 1.13)	<0.001
	Model III	1.11 (1.06, 1.15)	<0.001
	Model IV	1.11 (1.06, 1.16)	<0.001
ePVS_discharge	Unadjusted Model	1.34 (1.28, 1.40)	<0.001
	Model I	1.33 (1.28, 1.39)	<0.001
	Model II	1.17 (1.11, 1.23)	<0.001
	Model III	1.15 (1.09, 1.22)	<0.001
	Model IV	1.14 (1.08, 1.20)	<0.001

^1^n(%); Median (Q1, Q3).

^2^Pearson’s Chi-squared test; Kruskal-Wallis rank sum test.

**Figure 3:**
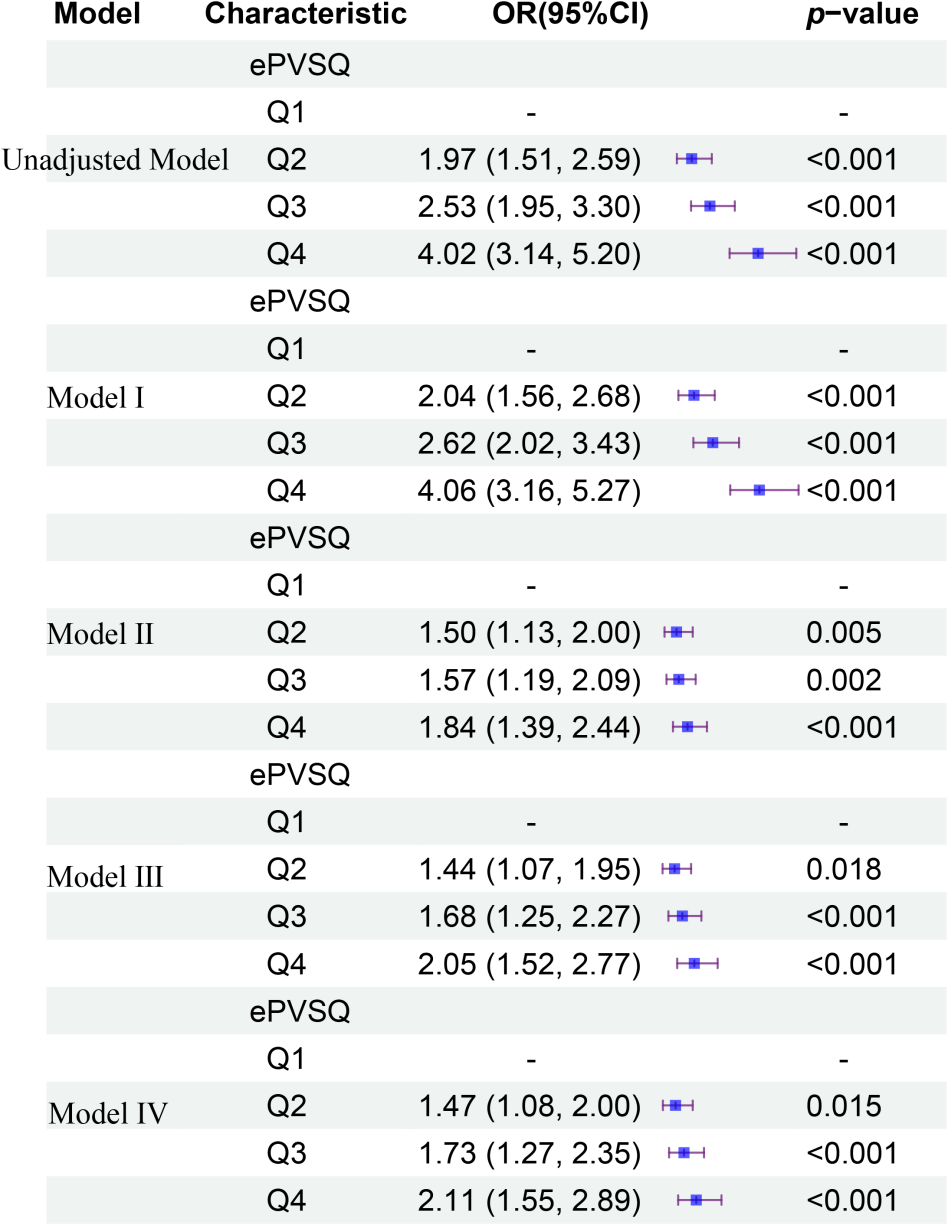
The relationship between different levels of ePVS and KTF in different models at admission.

The **Figure 3** provided a more detailed comparison of the relationship between different levels of ePVS and KTF in different models at admission. In the unadjusted logistic regression model, a high level of ePVS (Q4) was associated with an increased likelihood of KTF (OR 4.02; 95% CI 3.14-5.20; *p*< 0.001) compared to a low level of ePVS. We employed three logistic regression models to assess the association between ePVS and KTF in kidney transplant patients, adjusting for various confounding factors. In Model I, after adjusting for vital sign data (age, gender, LOS), a high level of ePVS (Q4) was associated with an elevated risk of KTF (OR 4.06; 95% CI 3.16-5.27; *p*< 0.001). In Model II, after further adjusting for comorbidities (HF, DA, CAD, HTN, AF, anemia, OSA) based on Model I, the high ePVS group continued to show a significantly higher risk of KTF (OR 1.84; 95% CI 1.39-2.44; *p*< 0.001). In Model III, after adjusting for drugs (tacrolimus, mycophenolate, furosemide, heparin, prednisone, warfarin, vancomycin, hydromorphone, oxycodone) based on Model II, the high ePVS group continued to demonstrated a significantly higher risk of KTF (OR 2.05; 95% CI 1.52-2.77; *p*< 0.001). Finally, In Model IV, after adjusting for laboratory data (INR, WBC, anion gap, calcium, chloride, and platelet) based on Model III, the high ePVS group still demonstrated a significantly higher risk of KTF (OR 2.11; 95% CI 1.55-2.89; *p*< 0.001). Therefore, following these analyses, we concluded that a high ePVS level was associated with an increased failure rate in kidney transplantation.

### Sensitivity and specificity analysis

**Figure 4:**
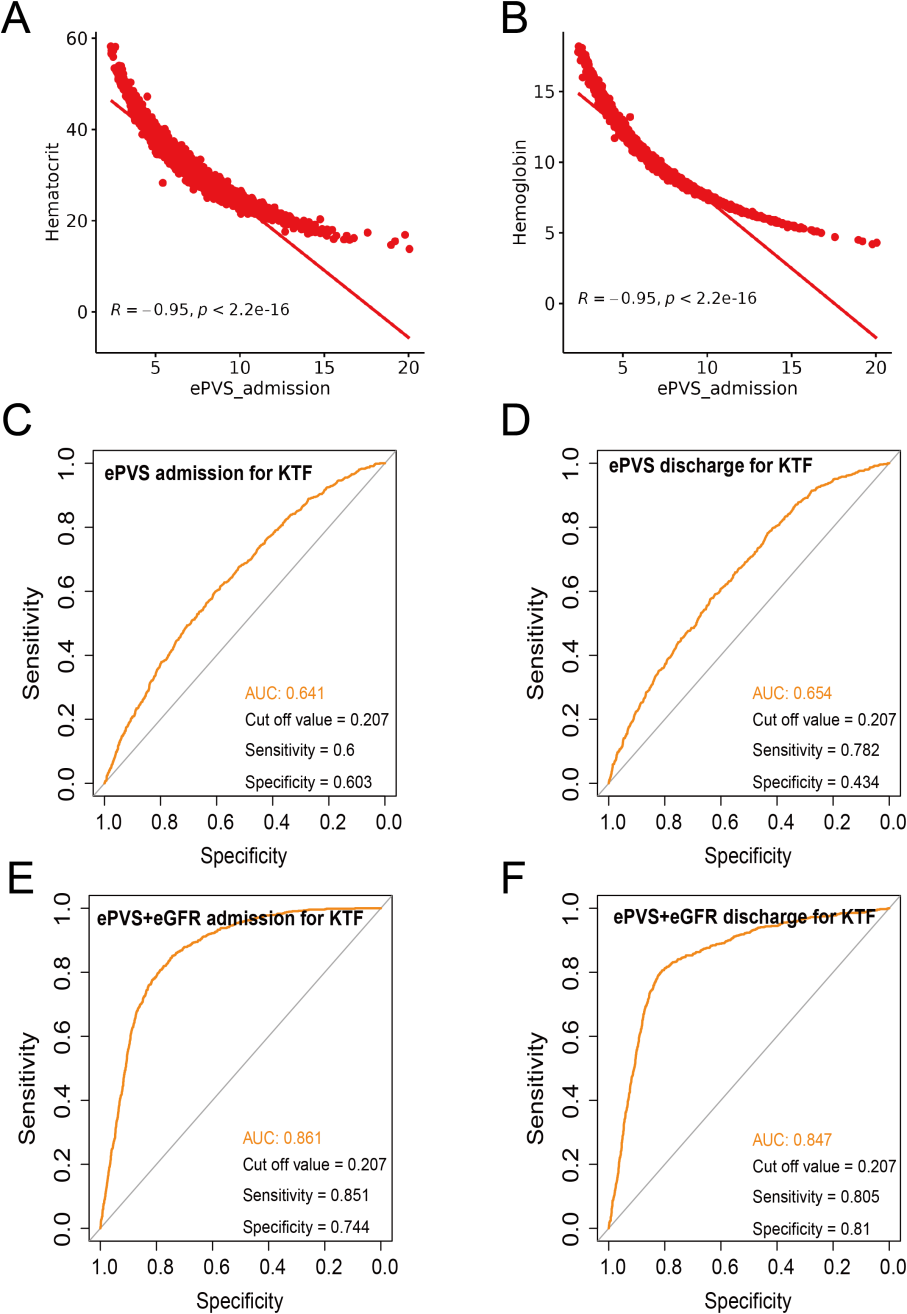
Sensitivity and specificity analysis. **(A)** Correlation analysis between ePVS admission and hematocrit. **(B)** Correlation analysis between ePVS admission and hemoglobin. **(C)** Sensitivity and specificity analysis for ePVS admission for KTF. **(D)** Sensitivity and specificity analysis for ePVS discharge for KTF. **(E)** Sensitivity and specificity analysis for ePVS+eGFR admission for KTF. **(F)** Sensitivity and specificity analysis for ePVS+eGFR discharge for KTF.

To support the correlation between ePVS and KTF, we performed sensitivity and specificity, as well as calculated the area under the ROC curve (AUC). First, the hematocrit and hemoglobin showed a strong negative correlation with ePVS (all R=-0.95, *P*<2.2e-^16^)(**Figures 4A, B**). Next, we separately analyzed the sensitivity and specificity of EPVs and KTF during admission and discharge. The sensitivity values were 0.6 and 0.782, the specificity values were 0.603 and 0.434, and the AUC values were 0.641 and 0.654, respectively, at admission and discharge (**Figures 4C, D**). Although it did not yield very satisfactory results, it still provided enough evidence to support our conclusions. We attempted to include eGFR in the analysis and the results showed a significant increase in sensitivity, specificity and AUC values, both at admission and discharge. Specifically, the sensitivity values were 0.851 and 0.805, the specificity values were 0.744 and 0.81, and the AUC values were 0.861 and 0.847, respectively, at admission and discharge (**Figures 4E, F**).

### Subgroup analyses

**Figure 5:**
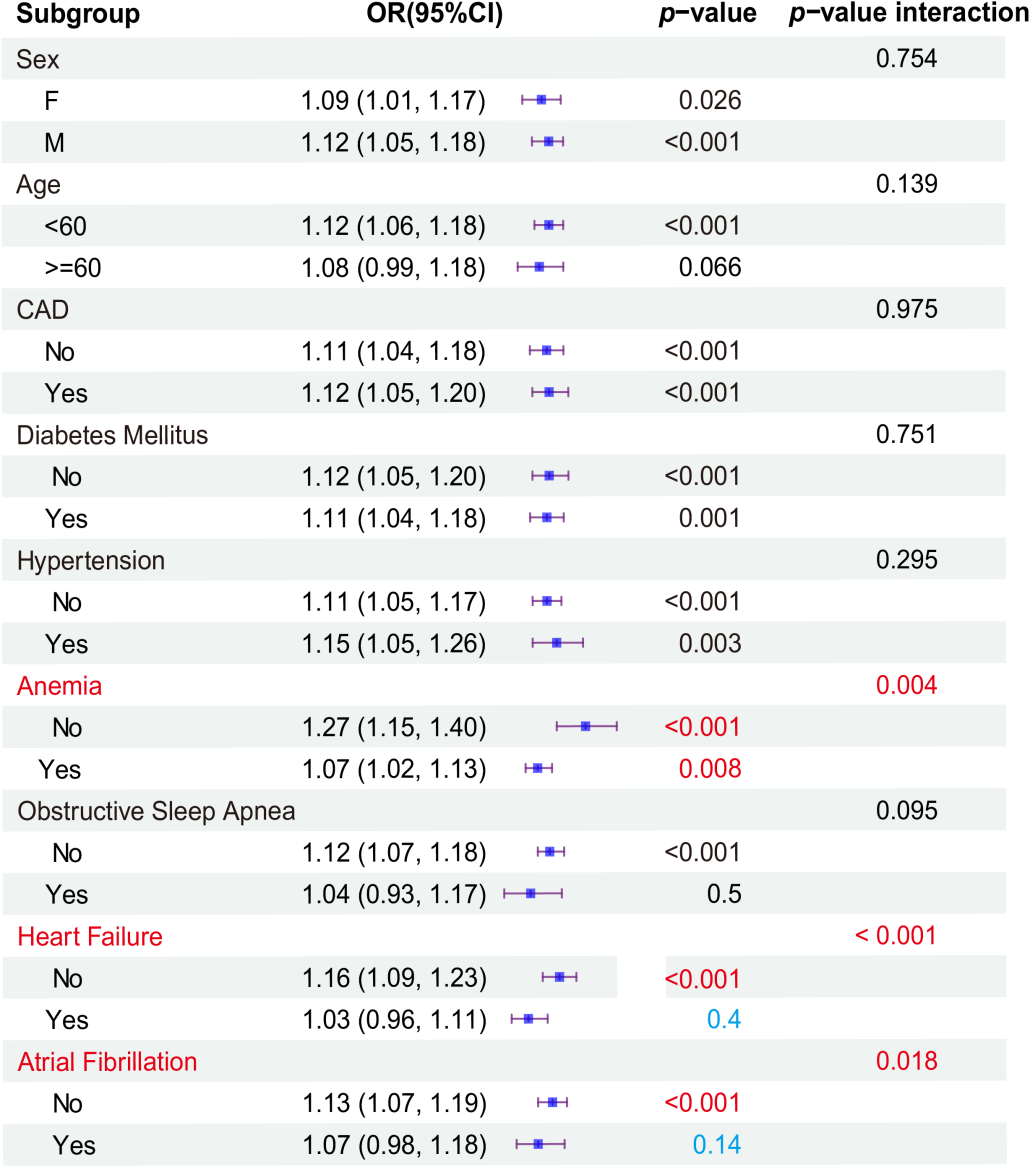
Subgroup analyses at admissions.

Subgroup analyses revealed significant associations within specific strata. At admissions (**Figure 5**), the male gender exhibited a heightened risk for the primary outcome, with an odds ratio (OR) of 1.12 (95%CI: 1.05, 1.18)(*P*<0.001), compared to female patients (OR: 1.09, 95%CI: 1.01, 1.17). Patients aged under 60 years demonstrated an HR of 1.12 (95%CI: 1.06, 1.18) (*P*<0.001). CAD and hypertension were at a significantly increased risk for the primary outcome (OR: 1.12, 95% CI: 1.05-1.20, *P*<0.001; OR: 1.15, 95% CI: 1.05-1.26, *P*=0.003) compared to Non-CAD (OR: 1.11,95% CI: 1.04, 1.18) and Non-hypertension (OR: 1.11, 95% CI: 1.05, 1.17). The patients with Non-DM (OR: 1.12, 95% CI: 1.05-1.20, *P*<0.001), Non-anemia (OR: 1.27, 95% CI: 1.15-1.40, *P*<0.001), Non-OSA(OR: 1.12, 95% CI: 1.07-1.18, *P*<0.001), Non-HF (OR: 1.16, 95% CI: 1.09-1.23, *P*<0.001), and Non-AF(OR: 1.13, 95% CI: 1.07-1.19, *P*<0.001) were associated with an elevated risk of the primary outcome compared to DM (OR: 1.11, 95% CI: 1.04-1.18), anemia (OR: 1.07, 95% CI: 1.02-1.13), OSA (OR: 1.04, 95% CI: 0.93-1.17), HF(OR: 1.03, 95% CI: 0.96-1.11), and AF (OR: 1.07, 95% CI: 0.98-1.18). To further validate the reliability of the results, interactive validation was used to analyze each subgroup. To further validate the reliability of the results, interactive validation was used to analyze each subgroup. The results showed a greater risk of KTF when ePVS was increased in Non-HF (*P* interaction< 0.001), Non-anemic (*P* interaction = 0.004), and Non-AF (*P* interaction = 0.018). However, when we analyzed the subgroups based on different levels of ePVS, only Non-HF (*P* interaction = 0.001) patients had a greater risk of KTF when ePVS was increased (**Supplementary Table S1**).

The results of the subgroup analysis of discharge were similar to admission, but there were differences. Similarly, age less than 60 years, being male, and with CAD had a higher risk for the primary outcome. Also, AF was associated with a higher risk of KTF than non-AF, unlike admission. The patients with Non-DM, Non-anemia, and Non-HF were associated with an elevated risk of the primary outcome compared to DM, anemia, and HF. Also, Non-hypertension was associated with a higher risk of KTF than hypertension, unlike admission. The specific results were shown in **Supplementary Figure S1**. Interaction validation showed a greater risk of KTF only when ePVS was increased in non-HF (OR: 1.17, 95% CI: 1.09-1.26, *P*< 0.001, *P* interaction=0.016).

## Discussion

ePVS was a marker of intravascular congestion, which indirectly reflects plasma volume shifts at the interstitial tissue level (2, 12). This study examined the association between ePVS and the risk of kidney transplant failure (KTF). A total of 4,421 kidney transplant patients were included, of which 3,661 (82.80%) had no kidney transplant failure (No-KTF) and 760 (17.20%) had kidney transplant failure (KTF). The mean age of all patients was 52.53 ± 13.00 years. The ePVS values exhibited a skewed distribution, with the admission patients concentrated in the range of 4–8 mL/g and the discharge patients concentrated in the range of 6–10 mL/g. From the RCS, we found that patients who have not yet undergone kidney transplantation may be more appropriate for evaluating the correlation between ePVS and KTF from RCS (**Figure 2**). The ePVS level in the KTF group (7.20 [5.78, 8.85]) was significantly higher than that in the non-KTF group (6.12 [4.95, 7.60]) (*p*< 0.001) at admission. High-level ePVS were accompanied by an increase in KTF risk (**Table 1**), and this association proved robust and independent of age, gender, and comorbidities. The ePVS may be a promising parameter for kidney transplant patients’ risk management (**Table 2**; **Figure 3**). Meanwhile, sensitivity and specificity tests also strengthened the association between EPVs and KTF (**Figure 4**).

Previous studies have suggested that ePVS could serve as a potential prognostic indicator for rehospitalization and mortality in patients with acute myocardial infarction (AMI) and heart failure (HF) (J. 13; X. 14–16). Elevated ePVS values have been linked to an increased risk of in-hospital mortality in acute AMI patients and those with right-sided heart failure (J. 13, 17). In our subgroup analysis, including interactive validation, we found that regardless of admission or discharge, the risk of KTF was greater when ePVS increased in Non-HF (*P*-interaction<0.001) (**Figure 5**).

This study examined the association between ePVS and the risk of kidney transplant failure (KTF). Our findings revealed that elevated ePVS was significantly associated with a higher risk of allogeneic kidney transplantation failure. This association proved robust and independent of age, gender, and comorbidities. The ePVS may be a promising parameter for kidney transplant patients’ risk management.

The prevalence of patients returning to dialysis after graft loss (DAGL) was expected to rise, as recent advancements in preventing early graft loss have not led to significant improvements in long-term outcomes (18). Moreover, there was a paucity of data to inform clinical practice concerning failing renal transplants, particularly in cases of advanced transplant-related chronic kidney disease nearing dialysis dependence. The mortality rate was significantly higher in patients who return to dialysis after graft loss (DAGL) compared to those awaiting their first transplant (19) or those who remain dialysis-independent, even with poor graft function (20, 21). Patients who return to dialysis have a 1-year mortality rate of 16% and a 3-year mortality rate of 33% (22). While kidney transplant failure is an independent risk factor for mortality (23), re-transplantation has been linked to an 88% reduction in mortality (24), leading many to advocate for strategies that minimize sensitization. Our study presents ePVS as a simple and efficient indicator for risk stratification in kidney transplant patients or those preparing for transplantation. However, further prospective clinical trials involving hematocrit/hemoglobin levels and ePVS are necessary to validate this hypothesis. In addition, there are still shortcomings in our research, such as in the clinical analysis of kidney transplantation, where donor type (live versus decoyed) and population reactive antibody (PRA) levels are key factors affecting transplant success rate and long-term prognosis. Due to database limitations, we were unable to further include these analyses. It is recommended to conduct multivariate regression analysis in real-world research, such as including donor type (*in vivo*/deceased), PRA level, cold ischemia time, donor age, etc., to predict the risk of rejection or DGF after transplantation. And utilize machine learning to integrate clinical and immunological data for personalized evaluation of transplant feasibility.

## Conclusion

In this study, we found that higher ePVS values, calculated simply from Duarte’s formula (based on hemoglobin/hematocrit), were accompanied by an increase in KTF risk, and this association proved robust and independent of age, gender, and comorbidities. Additionally, in our subgroup analysis, including interactive validation, we found that regardless of admission or discharge, the risk of KTF was greater when ePVS increased in non-heart failure. Therefore, ePVS may be an important reference parameter for kidney transplant patients and help improve risk stratification.

## Data Availability

The datasets presented in this study can be found in online repositories. The names of the repository/repositories and accession number(s) can be found below: https://physionet.org/content/mimiciv/3.1/.

## Supplementary material

Supplementary Figure 1 Subgroup analyses at discharges.
