# Supplementary figures and images for: High-level ePVS was accompanied by an increase in kidney transplant failure risk: analysis based on the MIMIC-IV database
Source: Front Immunol. 2025 Aug 29;16:1574525. doi: 10.3389/fimmu.2025.1574525 (PMC12425785; doi:10.3389/fimmu.2025.1574525)

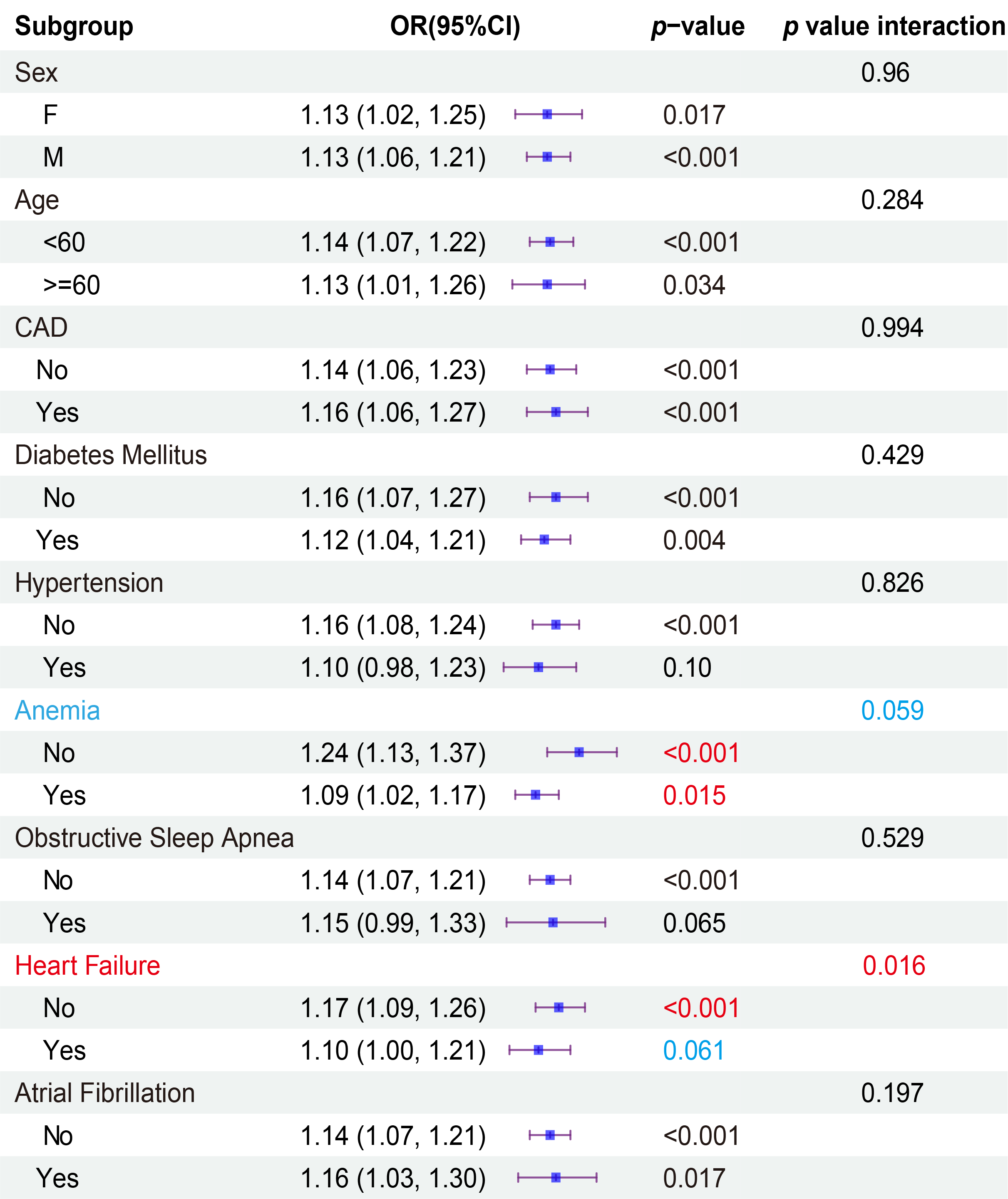

Supplement: Supplementary Figure 1 — Subgroup analyses at discharges. [file Image1.tif]
