# Supplementary material for: High-level ePVS was accompanied by an increase in kidney transplant failure risk: analysis based on the MIMIC-IV database
Source: Front Immunol. 2025 Aug 29;16:1574525. doi: 10.3389/fimmu.2025.1574525 (PMC12425785; doi:10.3389/fimmu.2025.1574525)
Supplement: Supplementary file 2 [file Table1.docx]

**Tab S1. Subgroup analysis of admission patients based on different ePVS levels**

| Subgroup | ePVS | OR(95%CI) | *p*-value | *p* value interaction |
| --- | --- | --- | --- | --- |
| Sex |  |  |  | 0.508 |
| F | Q1 | - | - |  |
|  | Q2 | 1.29 (0.74, 2.30) | 0.4 |  |
|  | Q3 | 1.19 (0.68, 2.13) | 0.5 |  |
|  | Q4 | 1.61 (0.92, 2.91) | 0.10 |  |
| M | Q1 | - | - |  |
|  | Q2 | 1.48 (1.01, 2.16) | 0.042 |  |
|  | Q3 | 1.96 (1.36, 2.85) | <0.001 |  |
|  | Q4 | 2.24 (1.54, 3.29) | <0.001 |  |
| Age |  |  |  | 0.697 |
| <60 | Q1 | - | - |  |
|  | Q2 | 1.30 (0.90, 1.89) | 0.2 |  |
|  | Q3 | 1.56 (1.08, 2.26) | 0.019 |  |
|  | Q4 | 1.90 (1.31, 2.76) | <0.001 |  |
| ≥60 | Q1 | - | - |  |
|  | Q2 | 1.94 (1.09, 3.56) | 0.027 |  |
|  | Q3 | 2.25 (1.27, 4.10) | 0.007 |  |
|  | Q4 | 2.58 (1.43, 4.81) | 0.002 |  |
| CAD |  |  |  | 0.704 |
| No | Q1 | - | - |  |
|  | Q2 | 1.70 (1.12, 2.59) | 0.013 |  |
|  | Q3 | 1.83 (1.21, 2.79) | 0.005 |  |
|  | Q4 | 2.28 (1.50, 3.50) | <0.001 |  |
| Yes | Q1 | - | - |  |
|  | Q2 | 1.25 (0.78, 2.03) | 0.4 |  |
|  | Q3 | 1.67 (1.05, 2.69) | 0.032 |  |
|  | Q4 | 2.06 (1.29, 3.33) | 0.003 |  |
| Diabetes Mellitus |  |  |  | 0.86 |
| No | Q1 | - | - |  |
|  | Q2 | 1.72 (1.07, 2.83) | 0.028 |  |
|  | Q3 | 2.02 (1.25, 3.30) | 0.004 |  |
|  | Q4 | 2.37 (1.46, 3.91) | <0.001 |  |
| Yes | Q1 | - | - |  |
|  | Q2 | 1.36 (0.91, 2.06) | 0.14 |  |
|  | Q3 | 1.58 (1.06, 2.38) | 0.027 |  |
|  | Q4 | 2.11 (1.40, 3.20) | <0.001 |  |
| Hypertension |  |  |  | 0.099 |
| No | Q1 | - | - |  |
|  | Q2 | 1.61 (1.12, 2.31) | 0.010 |  |
|  | Q3 | 1.57 (1.10, 2.27) | 0.014 |  |
|  | Q4 | 2.28 (1.59, 3.28) | <0.001 |  |
| Yes | Q1 | - | - |  |
|  | Q2 | 1.08 (0.58, 2.03) | 0.8 |  |
|  | Q3 | 2.13 (1.17, 3.95) | 0.014 |  |
|  | Q4 | 1.69 (0.89, 3.28) | 0.11 |  |
| Anemia |  |  |  | 0.119 |
| No | Q1 | - | - |  |
|  | Q2 | 1.86 (1.17, 2.98) | 0.009 |  |
|  | Q3 | 2.73 (1.70, 4.41) | <0.001 |  |
|  | Q4 | 2.74 (1.52, 4.89) | <0.001 |  |
| Yes | Q1 | - | - |  |
|  | Q2 | 1.15 (0.75, 1.78) | 0.5 |  |
|  | Q3 | 1.21 (0.80, 1.84) | 0.4 |  |
|  | Q4 | 1.59 (1.07, 2.40) | 0.023 |  |
| Obstructive  Sleep Apnea |  |  |  | 0.195 |
| No | Q1 | - | - |  |
|  | Q2 | 1.60 (1.13, 2.28) | 0.009 |  |
|  | Q3 | 1.87 (1.33, 2.66) | <0.001 |  |
|  | Q4 | 2.39 (1.69, 3.42) | <0.001 |  |
| Yes | Q1 | - | - |  |
|  | Q2 | 0.92 (0.45, 1.89) | 0.8 |  |
|  | Q3 | 1.38 (0.67, 2.85) | 0.4 |  |
|  | Q4 | 1.10 (0.52, 2.33) | 0.8 |  |
| Heart Failure |  |  |  | 0.001 |
| No | Q1 | - | - |  |
|  | Q2 | 1.83 (1.21, 2.77) | 0.004 |  |
|  | Q3 | 1.98 (1.31, 3.01) | 0.001 |  |
|  | Q4 | 3.06 (2.03, 4.68) | <0.001 |  |
| Yes | Q1 | - | - |  |
|  | Q2 | 1.00 (0.61, 1.65) | >0.9 |  |
|  | Q3 | 1.37 (0.85, 2.23) | 0.2 |  |
|  | Q4 | 1.17 (0.71, 1.93) | 0.5 |  |
| Atrial Fibrillation |  |  |  | 0.223 |
| No | Q1 | - | - |  |
|  | Q2 | 1.49 (1.03, 2.15) | 0.034 |  |
|  | Q3 | 1.81 (1.26, 2.61) | 0.001 |  |
|  | Q4 | 2.26 (1.57, 3.28) | <0.001 |  |
| Yes | Q1 | - | - |  |
|  | Q2 | 1.16 (0.62, 2.20) | 0.6 |  |
|  | Q3 | 1.46 (0.78, 2.77) | 0.2 |  |
|  | Q4 | 1.63 (0.87, 3.13) | 0.13 |  |
